# Progress in Medicine

**Published:** 1898-07-23

**Authors:** 


### PROGRESS IN MEDICINE.

### RENAL MEDICINE.

The frequency with which cases of granular kidney are overlooked is a most important matter and one to which Dr. Samuel West did well to draw the attention of the Harveian Society.[1] He pointed out that the reason usually is that there is little in the symptoms to draw attention to the kidney at all. The disease may become prominent during middle life, but has often existed long before, even from childhood. Acute nephritis in the adult is often a consequence of granular kidney of old standing and rarely a cause of it. Hæmorrhages of various kinds, such as epistaxis, recurrent hæmaturia, heart failure, and pericarditis, as well as general failure of vision may be the first noticeable signs of the disease. In fact, the patient usually comes under treatment for something which has little apparent relation to chronic nephritis, when a careful examination suddenly discloses that the supposed healthy individual has advanced kidney disease. How many cases of dyspepsia, vomiting, and even of ordinary diarrhœa are treated with no result until it is all at once found out that they are but the symptoms of granular kidney. Various skin eruptions may also be merely symptoms of this insidious disease; and among the nerve affections we find cramps, headaches with or without optic neuritis, or even peripheral neuritis, or apoplectiform attacks. Such then are the affections which may be the first signs of a fatal kidney lesion. R. Maguire, in the discussion on this paper, argued that granular kidney is not a cause of the various phenomena but one of the many effects of a universal degeneration of the tissues, and claimed that instances had been found where kidneys were normal, although arterial degeneration and very high tension were present. But even if we accept this revived theory once more, it is not less important to recognise the signs of a general disease which West has pointed out Indeed West himself elsewhere admits that the vascular changes are probably primary, due to some unknown cause, and preceding the disease in the kidney.[2]

Contracted kidney in children has been much discussed of late, and L. Bernard[3] finds that some of these cases are not connected with vascular changes, but that partial contraction may result from glomerular disease, the result of acute nephritis. Besides acute diseases, syphilis, absorption of toxins during intestinal disorders and calculi may be among the causes. Senator[4] holds that chronic nephritis is usually due to a faulty state of the blood. It may arise from acute nephritis and end in a secondary form of granular atrophy, or the poisons may cause a primary granular interstitial atrophy, or the latter disease may be due sometimes to vascular changes, sometimes even to a congenital stenosis of the renal vessels. Councilman[5] recognises an acute interstitial nephritis not dependent on a previous epithelial affection. The cells are chiefly derived from the endothelium of the vessels. Instances occur in scarlatina, diphtheria, and pyæmia, and a streptococcus was found in the cases he examined.

T. Rose Bradford[6] has observed a peculiar and definite type differing from granular kidney disease, and also from the large or small white kidney forms, which it may be useful to remember. There is a remarkable absence of dropsy, but the cases terminate in very acute uræmia, and are found chiefly in young women. The kidneys are small, averaging three ounces each, the capsule strips easily, but the surface of the kidney is very granular, and both it and the cortex are mottled in colour unlike that of the white kidney. Albuminuric retinitis is generally present. The urine is copious, the specific gravity low, but a large quantity of albumen is present. Finally, there is no reason to connect them with scarlatina or an acute nephritis complicating ordinary cirrhosis. Indeed, the first symptoms are trivial though death follows rapidly from uræmia.

**Acute Nephritis in Children.**—Dupeu[7] finds that this is extremely common, and much more hopeful than in adults. Measles, chicken pox, vaccinia, eczema, and any severe skin irritation such as that from burns, very hot baths, or strong carbolic lotion may produce it, and so may intestinal absorption in gastric disorders, especially where dilation of the stomach is present. Comby,[8] too, recognises the frequent occurrence of these mild attacks from simple infectious disorders, such as slight sore throat. A case of recurrent tonsillitis is mentioned by Dupeu, which was regularly followed by an attack. Comby thinks that a slight chill is often the first step to a bacterial invasion, which may be aided by tonsillitis, after which a pneumonia or nephritis develops. In these cases the ordinary signs of acute nephritis may appear, but there is a uniform rapid recovery. The question arises whether there is here a special weakness of the kidneys, and whether these are the patients who are later on found to be suffering from intermittent albuminuria.

**Uræmia.**—G. F. Still[9] discusses the death of an infant of two months from uræmia produced by cystic disease of the kidneys, where the liver was similarly affected. He believes that both conditions are due to developmental failure, but Rolleston refers them to interstitial inflammation, and notices that a similar state of the pancreas and brain had been observed. Whatever explanation of cystic kidney is adopted, it must be applicable to the disease in different organs. Monari[10] has advanced an important theory of uræmia. He finds that after excision of the kidneys or ligature of the ureters an invasion of various microbes occurs, and he thinks that in uræmia the bactericidal power of the blood is lessened, and that staphylococci, the B. coli, and other microbes gain an entrance and produce the toxic symptoms known as uræmic. C. E. Nammack,[11] in discussing the uræmia of acute exacerbations of chronic nephritis, collects many reports of beneficial venesections. This treatment, combined with injection of saline solution into the veins, tissues, or rectum, is the most hopeful we possess, and is now the routine method in some of the best American hospitals. T. Rose Bradford[12] distinguishes carefully between acute and latent uræmia. The latter occurs in suppression of urine both from obstruction, and also from sudden interference with healthy kidneys by shock, toxins, or thrombosis of renal arteries. In such cases we must be on our guard against advising surgical interference for a supposed calculus. There may be few or none of the ordinary symptoms of uræmia, but the patient generally dies after some days' suppression. On the other hand suppression, partial or complete, which comes on during Bright's disease, is followed by typical uræmia, either acute or chronic. Nervous symptoms are most prominent in the acute form, while gastro-intestinal ones are more marked in chronic cases; but both in these and the latent type there is a fall of temperature, except where some complication or numerous convulsions occur. The writer does not believe the retention theory, but points out that there is greatly increased and irregular metabolism in the muscles which possibly supply the toxic agent. Monari would, as we have seen, explain this as the result of a bacterial invasion. Bradford, from numerous experiments,[13] shows that excision of a considerable part of the kidney is followed by increased excretion of urea as well as of water, and that this increase of urea goes on even when no food is taken, so that there is an increased production of urea from a loss of control normally exercised by the kidneys over the tissues. Whether this is by an internal secretion similar to that of the thyroid is not clear, but against such a view we may urge that ligature of the ureters causes an increase just as excision of the whole organ does. The increased excretion of water after excision of perhaps two-thirds of the kidney is parallel to what is seen in granular kidney where the increase is probably proportional to the destruction of kidney substance rather than to changes in tension of the arteries. The absence of an increased excretion of urea in this disease may be due to the diseased character of the tubular epithelium. Finally, he shows that in suppression there is a great increase of urea and of creatin products in the blood and tissues, and a still greater one in acute uræmia without dropsy. In chronic renal disease there is some increase, but in eclampsia only to a trivial degree.

[1] Lancet, Mar. 5. [2] Clin. Jour., Feb. 23. [3] Charlotte Med. Jour., Feb. [4] Brit. Med. Jour., Jan. 29. [5] Brit. Med. Jour., April 23. [6] Practitioner, April. [7] Brit. Med. Jour., Dec. 11. [8] Med. Bulletin, Feb. [9] Brit. Med. Jour., Dec. 2. [10] Brit. Med. Jour., Mar. 26. [11] Med. Rec., Feb. 26. [12] Clin. Jour., April 20, and Lancet, April 2. [13] Lancet, Mar. 10.

